# Effect of zeolite (clinoptilolite) as feed additive in Tunisian broilers on the total flora, meat texture and the production of omega 3 polyunsaturated fatty acid

**DOI:** 10.1186/1476-511X-11-35

**Published:** 2012-03-06

**Authors:** Zouhir Mallek, Imen Fendri, Lamia Khannous, Amal Ben Hassena, Al Ibrahim Traore, Mohamed-Ali Ayadi, Radhouane Gdoura

**Affiliations:** 1 Centre Vétérinaire de Recherche, Sfax, Tunisia; 2 Unité de recherche Toxicologie - Microbiologie Environnementale et Santé (UR11ES70), Faculté des Sciences de Sfax, Université de Sfax-Tunisia, Sfax, Tunisia; 3 Laboratoire national de santé publique, Ouagadougou, Burkina Faso; 4 Laboratoire d'Analyses Alimentaires, Ecole Nationale d'Ingénieurs de Sfax, Université de Sfax, Sfax, Tunisia; 5 Département des Sciences de la Vie, Faculté des Sciences de Sfax, Rue de Soukra Km 3,5, BP 1171-3000 Sfax, Tunisie

**Keywords:** Zeolite, Weight of chicken, Organoleptic parameters, Omega 3 fatty acid

## Abstract

**Background:**

Increasing consumer demand for healthier food products has led to the development of governmental policies regarding health claims in many developed countries. In this context, contamination of poultry by food-borne pathogens is considered one of the major problems facing the progress of the poultry industry in Tunisia.

**Result:**

Zeolite (Clinoptilolites) was added to chicken feed at concentrations 0,5% or 1% and was evaluated for its effectiveness to reduce total flora in chickens and its effects on performance of the production. The broilers were given free and continuous access to a nutritionally non-limiting diet (in meal form)that was either a basal diet or a' zeolite diet' (the basal diet supplemented with clinoptilolite at a level of 0,5% or 1%). It was found that adding zeolite in the broiler diet significantly (*p *< 0,05) reduced total flora levels, as compared to the control, on the chicken body. In addition, it was found that zeolite treatment had a positive effect on performance production and organoleptic parameters that were measured and mainly on the increase level of Omega 3 fatty acid.

**Conclusion:**

This study showed the significance of using zeolite, as a feed additive for broilers, as part of a comprehensive program to control total flora at the broiler farm and to increase level of Omega 3 fatty acid on the chicken body.

## Background

Zeolites represent a large and very diverse group of minerals such as water-silicates that are characterized by three-dimensional structure and belong to the class of aluminosilicates from a chemical point of view. Their structure is based on a three-dimensional skeleton consisting of SiO_4 _and AlO_4 _tetrahedrons that form interconnected channels and cavities containing weakly bound (quite mobile) water molecules and cations of alkali metals (Na, K, Li, Cs) and alkaline earth metals (Ca, Mg, Ba, Sr), which compensate for the unsaturated negative valence of AlO_4 _[1]. Natural zeolites are hydrated aluminosilicates that have ion-exchange and adsorption properties and have a large surface area that helps in these adsorption properties [2,3].

The dietary use of naturally occurring or synthetic Zeolites has been reported to improve feed efficiency, thus leading to a beneficial growth response in broilers. Zeolite has been recommended and used effectively in reducing toxic effects of materials such as aflatoxins [4,5]. There is evidence that zeolite can be used as an antimicrobial agent [6]. Clinoptilolites were shown to be highly effective with regard to the metabolic utilization of nitrogen in poultry and pigs. This may indicate that one may decrease the concentration of nitrogen-containing substances in a feeding dose without affecting performance of animals [1,7].

Forms of zeolite have been used to remove ammonium from aqueous solutions [8] and help in reducing ammonia production by pullets and laying hens [9] and in broiler houses [10]. It is important to remove ammonia from poultry houses since it has been reported that high atmospheric ammonia in poultry facilities has been linked to damage the respiratory tract lining, reduced resistance to respiratory diseases, increased ascites and lower performance [11].

Fatty acid profiles in muscle and adipose tissue are affected by type of feed or forage, biohydrogenation kinetics, and turnover of fat [12]. Residual effects of diets and feeding regimes have been less researched however. A higher content of conjugated linoleic fatty acid (C18:2 cis-9-trans-11; CLA) in foods and lower omega 6 polyunsaturated fatty acid (n-6)/omega 3 polyunsaturated (n-3) fatty acid ratio have been shown to be beneficial to human health [12,13]. In this regard, dietary flax supplementation has been used by several authors in order to increase omega-3 (n-3) fatty acid content in beef [14]. Nevertheless, the effect of zeolite on omega-3 (n-3) fatty acid content is studied for the first time in this work.

Contamination of poultry by food-borne pathogens is considered one of the major problems facing the progress of the poultry industry in Tunisia. The main aim of the presented work was to investigate the effects of using different levels of natural zeolite, as a broiler feed additive, on reducing total flora in Tunisian broilers and to study whether these treatments would also have an overall beneficial effect on broiler production performance and on Omega3 level in thigh.

## Materials and methods

### Broiler management

The experiment was performed in the accredited experimental enclosure of Veterinary Research Centre, in Sfax-Tunisie. The animal protocols were approved by the Animal Research Panel of the Committee on Research Practice of the Sidi Thabet Veterinary School in Tunisia.

A total of 200 sexed one-day-old chickens (HubbardJV) were selected and divided into three groups: control group and two experimental groups (0,5% zeolite and 1% zeolite), each consisting of 67, 66 and 67 chickens respectively. Chickens were reared individually in aviaries equipped with a manual system of watering and feeding, in a setting with controlled light and temperature regime. Indeed, they were placed in a deep pit house, ventilated both naturally and mechanically, and illuminated both artificially and naturally through windows. Feed and water were provided as *ad libitum*. The experiment was finished when the chickens reached the age of 45 days.

Chickens received the complete feed mixture CF1 from the start of the experiment until day 10, followed by the feed mixture CF2 from day 11 to day 45. All feed mixtures contained the same components (Table 1); the only difference was that the mixtures designed for the experimental groups (0,5% zeolite and 1% zeolite) were supplemented with 0,5% and 1% of clinoptilolite (commercial additive ZeoFeed) to replace respective portions of wheat. Feed mixtures and drinking water were provided *ad libitum*. The levels of basic components and nutrient composition of feed mixtures are provided in Table 1.

**Table 1:** Composition of feed mixtures (%) and the content of basic nutrients (g/kg)

Nutritional values of CF1 diet	
**Nutrients**	**%**

Protein	19

Fat	3

Energy (Kcal/kg)	2900

Crude cellulose	5

P	0,6

Ca	1

Ash	7



**Nutritional values of CF2 diet**	

**Nutrients**	**%**

Protein	17

Fat	3

Energy (Kcal/kg)	3000

Crude cellulose	4

P	0,6

Ca	1

Ash	6

### Active substance specification

The feed additive ZeoFeed which was used in this experiment contains at least 84% of clinoptilolite as an active substance, at a moisture level of max. 6%. It also contains 65% of SiO_2_, 12% of Al_2_O3, 2.3% of Fe_2_O3 and 2.7% of CaO (Table 2). Particle size varies in a range of 0,2-0,5 mm.

**Table 2:** Semi-quantitative mineralogical composition and chemical microanalysis of the natural zeolite, which was added in the diet

Chemical Component	%WT
**SiO2**	65-71.3

**Al2O3**	11.5-13.1

**Ca O**	2,7-5,2

**K2O**	2,2-3,4

**Fe2O3**	0,7-1,9

**Mg O**	0,6-1,2

**Na2O**	0,2-1,3

**TiO2**	0,1-0,3



**Mineralogical Component**	**% WT**

**Clinoptilolite**	84

**Cristobalite**	8

**Feldspar**	4

**Illite**	4

**Quartz**	Traces

**Carbonated minerals**	< 0,5

### Total flora enumeration

Enumeration of total flora is made by the reference method (ISO4833) for the enumeration of microorganisms as described by Feldsine et al. [15]. This method is used in alimentary bacteriology to enumerate aerobic bacteria in some food products. 25 g of sample (broiler intestine) was mixed in 225 ml of buffered peptone water. Appropriate decimal dilutions were performed and 1 ml of each required dilution was then transferred into a Petri dish with 15 ml of tempered PCA agar. When solidified, Petri dishes were incubated at 30 ± 1°C for 72 h ± 3 h (detection of mesophilic bacteria). Only plates containing less than 300 colonies but greater than 10 were used for enumeration.

### Live weight determination

The weight of the chickens was determined every week on Days 1, 8, 15, 22, 29, 36, and 45 of the chickens' age. The weight was determined at the accuracy of the tenth of a gram, at the same time of day and at the same order of groups. Mortality in each group was also recorded when occurred.

### Intramuscular moisture, protein, ash, fat and fatty acid composition

At the end of the experiment, the chickens were slaughtered. Nine thighs of each group were randomly selected and harvested. The muscle portion was cut into 2 steaks and used for determination of moisture, protein, ash, intramuscular fat (IMF) and fatty acid (FA) composition. Duplicate 5-g samples of muscle were analyzed for nitrogen content in a Tecator apparatus and multiplied by 6.25 to determine crude protein (CP) content (Method 981.10) [16]. Moisture was determined by weight loss after drying the thigh sample (in 5-g duplicates) at 100°C for 24 h. Total ash was determined by ashing the sample at 600°C for 8 h (Method 920,153) [16].

Intramuscular fat (IMF) was determined by extracting the lipid fraction from 10-g aliquots of the muscle with solvent (hexane) in a Tecator apparatus (Method 991.36) [16]. In preparation, samples were trimmed of all external fat, and minced in a blade grinder.

Total lipids for fatty acid analysis were extracted from 5-g aliquot samples of thigh according to Folch et al. [17-19].

Fatty acid methyl esters (FAME) were prepared according to Pariza et al. [20], and measured by gas chromatography of FAME in a Chrompack CP 900 apparatus fitted with a flame ionization detector. Analytical gas chromatography was carried out on a Hewlett-Packard 6890 gas chromatograph series II (Agilent Technologies, Palo Alto, California, USA) equipped with HP Innowax (30 m × 0.25 mm, 0.25 µm film thickness) capillary column. Each sample was injected with a split ratio of 1:100 and a continuous flow rate of 1.5 ml/min of chromatographic grade helium was used. The oven temperature was initially held for 20 min at 165°C, ramped at 5°C/min up to 240°C and held isothermal for 25 min. Injector and FID detector temperature were held at 250°C. FAMEs were identified by comparison of their retention time with respect to pure standard purchased from Sigma and analyzed under the same conditions. FAMEs were quantified according to their percentage area, obtained by integration of the peaks. The results were expressed as a percentage of individual fatty acids in the lipid fraction as described by Pordomingo et al. [13].

Main individual fatty acids were grouped in saturated fatty acids (SFA = -myristic (C14:0) + palmitic (C16:0) + stearic (C18:0)), monounsaturated fatty acids (MUFA = myristoleic (C14:1) + palmitoleic (C16:1) + oleic (C18:1)) and polyunsaturated fatty acids (PUFA = n-3 + n-6 fatty acids), n-3 fatty acids (linolenic (C18:3) + eicosapentaenoic (EPA; C20:5) + docosapentaenoic (C22:5; DPA) + docosahexaenoic (DHA; C22:6), n-6 fatty acids (linoleic (C18:2) + di-homo-gamma-linolenic (DGLA; C20:3) + arachidonic (AA; C20:4) + docosatetraenoic (adrenic; C22:4). In this study, only SFA, palmitoleic and oleic MUFA and linoleic and linolenic PUFA (among Omega 6 and Omega 3 groups respectively) were analyzed.

### Health

The health condition of broiler chickens was monitored throughout the experiment, with no clinical symptoms of any disease being recorded.

### Texture measurement

All instrumental texture analyses were done on thigh samples stored at least for 24 h at 4°C. For every formulation two repeated measurements were taken for each replicate and mean values are reported. Texture profile analysis (TPA) of thigh samples was performed [21]. Each sample was cut from the centre and compressed twice to 50% of their original height using a texturometer (Texture Analyser, TA Plus, LLOYD instruments, England). In these experiments hardness, elasticity and chewiness were determined.

### Statistical evaluation

All results are expressed as the mean ± standard deviation (± SD). The SAS System for Windows, V8 (SAS Institute, Gary, NC) was used for statistical evaluations. Means ± S.D. were calculated for normalizing the control as 100%. Differences among treatment and control groups were tested by one way analysis of variance (ANOVA), followed by pair-wise comparisons between group using Tukey's test. Differences at *p <*0,05 were considered significant.

## Results and discussion

### Effects of zeolite treatments on total flora reduction

**Figure 1:**
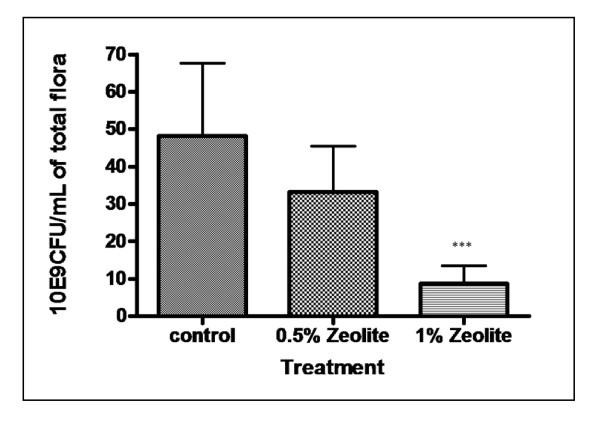
**Effects of different treatments on total flora prevalence in broiler intestine 25 g of sample was mixed in 225 ml of buffered peptone water**. Appropriate decimal dilutions were performed and 1 ml of each required dilution was then transferred into a Petri dish with 15 ml of tempered PCA agar. When solidified, Petri dishes were incubated at 30 ± 1°C for 72 h ± 3 h. Only plates containing less than 300 colonies but greater than 10 were used for enumeration.

The effects of adding different levels of Zeolite in the broiler feed on the prevalence of total flora at the end of experiment are shown in Figure 1. Our results show that adding Zeolite, at least at the higher dose (1%), significantly (*p *< 0,05) reduced total flora contamination on the intestine at the 45 day-of-age. The reduction was over 50% of the control group. It is important to note that an average reduction of more than 50% achieved by the 1% Zeolite treatment could contribute to the safety of poultry. The antimicrobial effect of Zeolite on *Salmonella *and other enteric bacteria reduction has been previously reported [22,23]. It is possible that the effects of Zeolite could be due a chelating properties or an intrinsic mechanism in the body that could lead to litter moisture reduction. Adding 2% Zeolite in the broiler feed, reduced litter moisture [24] and decreased the organic content of the litter and improved its quality [25]. The resulted dried litter leads to the destruction of different microorganisms. Different results were obtained using 0,5% (Figure 1) and 2% (data not shown) of Zeolite. In this case, the number of total flora is not significantly (*p *> 0,05) different from the control.

### Measurements of broiler performance indicators

Table 3 shows the average growth of broilers, at the end of the experiment, according to treatment groups. Average growth rates were significantly (*p *< 0,05) different between broilers of different groups; broilers that were fed on the 'zeolite diet' were growing to a faster rate (*p *< 0,05) compared with those of the control group. However, such differences were more evident after day 37 (data not shown) in each of the three rounds of the experiment.

**Table 3:** Effect of Zeolite in broiler diet on production performance

	control	0,5% zéolite	1% zéolite
Total fat (%)	2,27 ± 0,23	2,52 ± 0, 3	2.23 ± 0,3

Crude protein (%)	69,83 ± 2,35	67,74 ± 3,46	69,89 ± 1,74

Ash (%)	1,34 ± 0,35	1,56 ± 0,23	1,43 ± 0,17

Dry matter (%)	28,46 ± 2,03	29,15 ± 1,79	27,8 ± 0,81

Body weight (kg)	2,24 ± 0,12	2,17 ± 0,16	2,44 ± 0,10***

In fact, the administration of clinoptilolite (0,5 and 1% of the commercial additive ZeoFeed) in the feed mixtures for the chickens in the experimental group was positively reflected in performance indicators. Broilers fed the 1% zeolite diet were significantly heavier from those fed the basal diet at the age of 45 days (2,24 kg vs. 2,44 kg, *p *< 0,001). The corresponding differences become significant on Day 39 of the chickens' age *P <*0,001). The weight of chickens in the experimental group with 0,5% zeolite and at the end of the experiment was not significantly different (*P > 0,05*). These results agree with previous reports in which it was found that zeolite added in the broiler feed increased body weight [26] and increased growth rate [25]. This positive effect of zeolite on performance production could be due to its ability to reduce toxic effects of materials such as aflatoxins as has been reported in several studies [4,5].

Chickens were slaughtered at the end of the experiment, i.e. on Day 45. The thighs were collected and analyzed for the contents of moisture, protein, ash, intramuscular fat (IMF), organoleptic properties and fatty acid (FA) composition.

Total FA percentage measured in the thigh muscle was 2.27% of the total control thigh weight and was not significantly different (*p *> 0,05) compared with the other treatments at this sampling point (Table 3).

Crude protein content, moisture and total ash were also not affected (*p *> 0,05) by dietary treatments.

### Intramuscular fatty acid composition

It is essential to note that from the standpoint of vascular disease prevention, n-3 PUFAs are the most important and extensively studied class of essential PUFA. n-3 and n-6 PUFAs are termed "essential" FA and must be obtained from the diet because humans lack the Δ12- and Δ15-desaturases necessary to insert a double bond at the n-3 or n-6 position of an FA carbon chain. The difference between the two essential PUFA is based on the location of the first double bond of the molecule counting from the methyl end of the FA [13].

Increasing the n - 3 fatty acid content in thigh can be done by increasing the overall lipid content of meat or by increasing the percentage of n - 3 fatty acids in total fatty acids. No difference (*p *> 0,05) was observed in the total intramuscular fatty acid content among treatments (Table 4). Similar results were reported by Noci et al. [27] and Scollan et al. [28] when ~3% flax oil or ~4% whole flaxseed, respectively, were included in grass based (pasture and grass silage, respectively) diets.

**Table 4:** Effect of Zeolite dietary supplementation on intramuscular fatty acid composition (% total fatty acids)

Fatty acids (%)	control	0,5% zeolite	1% zeolite
**Saturated fatty acids (SFA)**			

Myristic acid (C14:0)	0,41 ± 0,02	0,30 ± 0,02 ^ad^	0,24 ± 0,02 ^ad^
Palmitic acid (C16:0)	21,28 ± 0,40	19,02 ± 0,15 ^a^	19,48 ± 0,28 ^a^
Stearic acid (C18:0)	5,50 ± 0,27	3,91 ± 0,32 ^a^	4,50 ± 0,32 ^b^

**Mono-unsaturated fatty acids (MUFA)**			

Palmitoleic acid (C16:1)	12,10 ± 0,21	9,18 ± 0,24 ^af^	7,42 ± 0,23 ^af^
Oleic acid (C18:1)	40,71 ± 0,26	43,14 ± 1,14 ^ac^	44,75 ± 0,31 ^ac^

**Poly-unsaturated fatty acids (PUFA)**			

Linoleic acid (C18:2)	19,40 ± 0,24	23,38 ± 0,36 ^ac^	22,77 ± 0,26 ^ac^
Linolenic acid (C18:3)	0,57 ± 0,01	1,03 ± 0,10 ^ae^	0,80 ± 0,08 ^be^

Each value is the mean of three determinations followed by standard deviation.

^a ^Significant differences between the control/0,5% Zeolite and control/1% Zeolite *p *< 0,05.

^b ^Significant differences between the control/0,5% Zeolite and control/1% Zeolite *p *< 0,01.

^c ^Significant differences between the control/0,5% Zeolite and control/1% Zeolite *p *< 0,001.

^d ^Significant differences between 0,5% Zeolite/1% Zeolite *p *< 0,05.

^e ^Significant differences between 0,5% Zeolite/1% Zeolite *p *< 0,01.

^f ^Significant differences between 0,5% Zeolite/1% Zeolite *p *< 0,001.

Feeding flaxseed has been shown to increase intramuscular fat levels of n - 3 fatty acids by several authors [29,30]. In the present experiment, addition of a natural mineral, the zeolite, was shown to increase significantly (*p *< 0,001) the level of total n - 3 fatty acids in intramuscular fat (Table 4), with this response being primarily related to higher levels of linolenic acid. Similar results were reported by Juarez et al. [14] using flaxseed and vitamin E. Authors showed no effect on intramuscular levels of 22:5n - 3 and 22:6n - 3 (*p *> 0,05). The lack of effect on 22:6n - 3 may be explained by the competition between 18:3n - 3 and the precursor for 22:6n - 3 (i.e. 24:5n - 3) for the activity of the Δ6 desaturase enzyme [21]. The increase of linolenic acid reached 200% when 0,5% of zeolite was added (Table 4). Moreover, an increase of the linoleic acid (C 18:2) was observed (*p *< 0,05). It is important to note that PUFAs (Linoleic Acid and Alpha-Linolenic Acid) have displayed protection against lipid peroxidation increasing the levels of several cellular antioxidants such as ascorbic acid, a-tocopherol and GSH [31].

Long chain fatty acid synthesis is controlled by a complex enzymatic system, consisting of desaturases and elongases. However, the degradation of these fatty acids is done by β-oxidation supplemented by two enzymes, reductase and isomerase. The increase of the n - 6 and n - 3 fatty acids in thigh muscle could be explained by a less degradation of these fatty acids by the intestinal microbes. Also, we can hypothesize that the enzymes responsible of fatty acids degradation could be down-regulated by some elements in zeolite.

The effects of zeolite on two monounsaturated fatty acids (MUFA) were showed in Table 4. Zeolite increases the percentage of 18:1 oleic acid and however decreased 16:1 palmitoleic acid percentage, again likely as a result of a redction in Δ9 desaturase activity. Similar results were reported by Juarez et al. [14].

Addition of Zeolite reduced clearly the level of total saturated fatty acids (SFA; pb0,001) in intramuscular fat (Table 4) and this was attributed to a reduction in 16:0 palmitic acid (*p *< 0,001), in 14:0 myristic acid (*p *< 0,001) and in 18:0 stearic acid. The increase of the level of unsaturated fatty acid mainly n-3 linolenic acid could be the direct result of the decrease level of SFA.

### Effect of zeolite addition on textural parameters of thigh muscle

**Figure 2:**
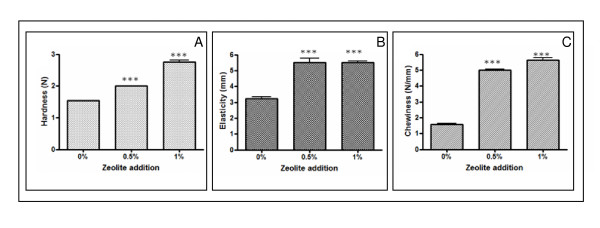
**Effect of Zeolite on organoleptic quality of thigh muscle**. **A**: Hardness (N), **B**: Elasticity (mm), **C**: Chewiness (N/mm). For every formulation two repeated measurements were taken for each replicate and mean values are reported.

Figure 2 shows the evolution of textural parameters of thigh muscle as a function of zeolite level added. Figure 2a presents evolution of the muscle hardness versus zeolite percentage added. From this figure we can conclude that, zeolite, mainly when added at 1% causes a significantly increase (*p *< 0,001) of thigh muscle hardness. Indeed, hardness of thigh muscle varied from 1,53 to 2,76N when zeolite concentration varied from 0% to 1%, respectively.

The evolution of thigh muscle elasticity with zeolite concentration is presented in Figure 2b. In accordance to hardness, zeolite addition has a significant effect (*p *< 0,05) on the elasticity of the muscle. Indeed, elasticity of thigh muscle varied from 3,23 to 5,5 mm when zeolite concentration varied from 0% to 1%, respectively.

Figure 2c shows thigh chewiness variation with zeolite concentration. As is the case with hardness and elasticity, this figure shows that chewiness increases with zeolite addition. All these textural changes can be explained in terms of the influence of the presence of natural zeolite on the gelling process of proteins. Zeolite-muscle proteins interaction leads to a change in texture and microstructure of the formulated thigh. Despite zeolite, the effect of other additives like carrageenan on the functional properties of meat products has been the subject of numerous studies [32]. In this case, Hsu and Chung [33] observed an increase in cooking yield, hardness, and other textural profile analysis parameters when adding up to 2% j-carrageenan to low-fat emulsified meatballs. Ruusunen et al. [34] and Garcia-Garcia and Totosaus [35], reported that addition of carrageenan lead to increased hardness in low-fat sausages products.

Such an evolution of textural parameters can be explained by the change in the water holding capacity. When zeolite was added, a reduction in the compactness of protein gel network allows more binding of water and makes meat tender.

## Conclusion

Natural materials, such as zeolite, are used as alternatives in poultry production systems due to their favorable effects upon growth and performance of broilers [25]. As expected, this work showed that the addition of zeolite in the diets decreased total flora in the intestine of broiler and led to an improvement of organoleptic quality of meat. Broilers fed the zeolite diet were significantly heavier from those fed the basal diet at the age of 45 days and were mainly enriched on intramuscular n - 3 fatty acid content. Several researches previously used flaxfeed in order to increase polyunsaturated fatty acids in meat. It is important to note that zeolite, the natural mineral, is cheaper than flax and so can be easily used in farms.

## Competing interests

The authors declare that they have no competing interests.

## Authors' contributions

ZM and IF designed the experiments, analyzed the data and drafted the manuscript. AB, LK and AT helped in data analysis. MA and RG conceived research and approaches and have given final approval of the manuscript to be published. All authors read and approved the final manuscript.
